# Advances and Future Directions in Hepatobiliary Malignancies

**DOI:** 10.3390/curroncol30110710

**Published:** 2023-11-07

**Authors:** Kojiro Taura

**Affiliations:** Division of Hepato-Biliary-Pancreatic Surgery and Transplantation, Department of Surgery, Graduate School of Medicine, Kyoto University, Kyoto 606-8507, Japan; ktaura@kuhp.kyoto-u.ac.jp

I am delighted to present this Special Issue of our journal, focusing on “Hepatobiliary Malignancies: Recent Advancements and Future Directions”. As the Guest Editor, it has been an honor to compile a diverse collection of papers that highlight significant developments in the field. This issue brings together cutting-edge research, clinical insights, and thought-provoking perspectives that contribute to the ongoing progress in managing hepatobiliary malignancies.

The papers featured in this issue encompass a wide range of topics, spanning from innovative treatment modalities to prognostic markers and advancements in imaging techniques. The authors have presented their findings and experiences with a level of expertise that will undoubtedly enhance our understanding and shape future directions in the field. I would like to briefly discuss a few notable contributions from this collection.

Radiation therapy is a crucial component in the management of hepatobiliary malignancies, and the paper on fiducial markers by Moskalenko M et al. demonstrates the importance of the accurate and reproducible delivery of liver stereotactic body radiation therapy. This study highlights how technological advancements have improved the precision and efficacy of radiation treatment, potentially leading to better outcomes for patients.

The association between osteopenia and survival in patients with intrahepatic cholangiocarcinoma provides insight on an intriguing aspect of the disease. The study by Miki A et al. emphasizes the importance of considering bone health as a prognostic factor, leading to a more comprehensive approach to patient care.

In the realm of surgical interventions, the proposal of laparoscopic hepatectomy for giant liver tumors presents a valuable perspective. Nitta H et al. suggest a safe and effective approach to managing these challenging cases, demonstrating the potential of minimally invasive techniques in hepatobiliary surgery.

The issue also covers important considerations in the context of systemic therapies, with papers discussing the efficacy of immune-based combinations and emerging systemic treatments for advanced unresectable biliary tract cancer. The studies by Kanai M, Rizzo A, and Tam VC provide valuable insights into the evolving landscape of systemic treatment options, paving the way for future developments in this area.

Additionally, we have included papers on topics such as biliary tract cancer follow-up programs, photodynamic therapies, and advances in local ablative therapies. These contributions expand our knowledge and understanding of various aspects of hepatobiliary malignancies, contributing to the overall goal of improving patient outcomes.

It is worth noting that this Special Issue reflects the global nature of research and clinical practice in hepatobiliary malignancies, with papers highlighting experiences from various institutions and countries. This diversity underscores the collaborative efforts and shared commitment of the scientific community to advance our understanding of these challenging malignancies.

I would like to express my gratitude to all the authors for their exceptional contributions to this Special Issue. Their dedication and expertise have made this collection a valuable resource for researchers, clinicians, and all those involved in the field of hepatobiliary malignancies.

Finally, I would like to thank the reviewers and the editorial team for their diligent work in ensuring the high quality of the papers presented in this issue. Their expertise and attention to detail have been instrumental in shaping this collection.

I hope that this Special Issue will serve as a catalyst for further research, collaboration, and innovation in the field of hepatobiliary malignancies. May it inspire and guide us towards improved diagnostic techniques, novel treatment strategies, and better outcomes for our patients.

In total, twenty manuscripts were accepted for publication and inclusion in this Special Issue. The contributions are listed in the List of Contributions.

